# Description of CRISPR-Cas9 development and its prospects in human papillomavirus-driven cancer treatment

**DOI:** 10.3389/fimmu.2022.1037124

**Published:** 2022-11-21

**Authors:** Yuhao Wei, Zhen Zhao, Xuelei Ma

**Affiliations:** ^1^ Department of Biotherapy, Cancer Center, West China Hospital, Sichuan University, Chengdu, Sichuan, China; ^2^ West China School of Medicine, West China Hospital, Sichuan University, Chengdu, Sichuan, China

**Keywords:** human papillomavirus, clustered regularly interspaced short palindromic repeat/CRISPR-associated nuclease 9, gene editing, cancer treatment, tumor microenvironment

## Abstract

Human papillomaviruses (HPVs) have been recognized as the etiologic agents of various cancers and are called HPV-driven cancers. Concerning HPV-mediated carcinogenic action, gene therapy can cure cancer at the molecular level by means of the correction of specific genes or sites. CRISPR-Cas9, as a novel genetic editing technique, can correct errors in the genome and change the gene expression and function in cells efficiently, quickly, and with relative ease. Herein, we overviewed studies of CRISPR-mediated gene remedies for HPV-driven cancers and summarized the potential applications of CRISPR-Cas9 in gene therapy for cancer.

## Introduction

Human papillomaviruses (HPVs) are nonenveloped epitheliotropic viruses with eight coding genes and a circular double-stranded DNA genome (1). HPVs are important human pathogens, and many viral diseases, such as cervical cancer caused by HPVs, have received immense attention as a result of their high transmission rates and difficulty in curing (2). During HPV-driven cancer development, viral DNA is frequently integrated into host cell chromosomes, and the proteins encoded by viral genes play a critical role in carcinogenesis (3, 4).

Collectively, HPV-driven cancers include cervical cancer, anal cancer, oral cancer, oropharyngeal cancer, and other cancers. In recent decades, many studies have reported various breakthroughs in the field of cervical cancer, with the earliest and deepest progress on the HPV-relevant mechanism. A further study explored the prevention and treatment of cervical cancer and used it as the breach point of other HPV-driven cancers (5).

The CRISPR-Cas9 system is an adaptive defense network in microorganisms and has become the leading technology for genome editing (6). Compared with other genome editing technologies, such as ZFNs and TALENs, it has the advantage of being programmable with short RNAs, which makes it easier to use (7). There is already a study indicating that CRISPR-Cas9 has a role in targeting DNA (8) and was subsequently proven to be able to edit human chromosomal DNA (9). Building on these studies, in 2020, a phase I clinical trial of CRISPR-Cas9 was completed in patients with advanced non-small cell lung cancer (10), which opened the beginning of the technology for the clinical treatment of oncology. In recent years, CRISPR-Cas9 has been increasingly used to target the HPV gene that induces most cervical cancer tumors. In 2014, the E6 and E7 genes of the HPV virus were targeted by using CRISPR-Cas9, leading to the death of tumor cells (11). This study provides a theoretical basis for the treatment of HPV-induced cancers. Studies on the stability and safety of CRISPR-Cas9 are still ongoing, as preclinical and clinical studies have been conducted.

In this review, we discuss the current state of development of CRISPR-Cas9 for HPV clinical treatment based on a summary of the theoretical rationale and relevant trials. We also highlight the potential application of this technology for HPV clinical treatment.

## Mechanism of CRISPR-Cas9 technology

**Figure 1:**
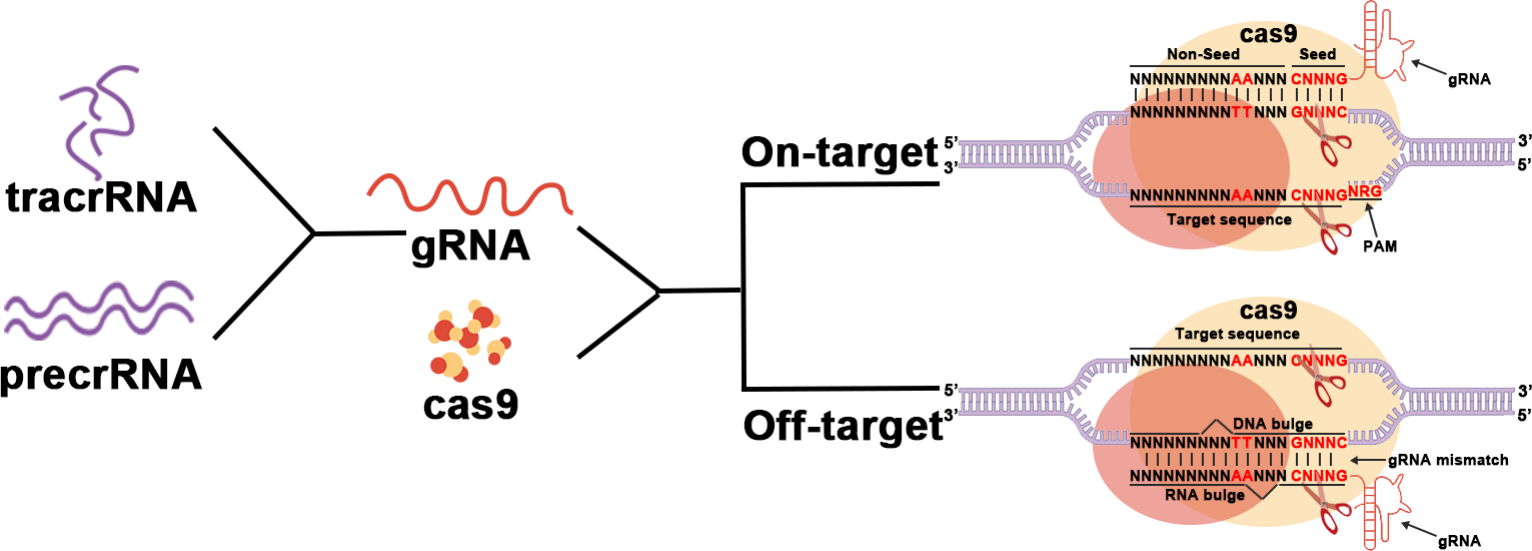
The main working procedure of CRISPR-Cas9.

CRISPR-Cas9 systems are divided into two primary classes. CRISPR-Cas9 belongs to type II in Class 2 (12). It consists of a CRISPR array in the middle and several Cas genes on both sides, including the specific cas9 gene. The CRISPR RNA transcribed by the CRISPR array (gRNA) acts to direct the nuclease Cas9 to generate a double-strand break (DSB) at the target site (13). (**Figure 1**)

First, after invading the host, exogenous DNA is cleaved into several DNA fragments called protospacers. Protospacers will then be inserted between the high-frequency repeat fragments to form the CRISPR array. The proteins Cas1 and Cas2 play an important role in this process (12, 14, 15). The Cas1 protein has been proven to have endonuclease activity and directs protospacer insertion between repetitive fragments (16). However, how the Cas2 protein works remains unclear.

Subsequently, the CRISPR array will be transcribed to generate precrRNAs that match the target gene. The CRISPR site located upstream of the CRISPR array is transcribed to generate tracrRNA. TracrRNA has a fragment homologous to precrRNA and therefore can bind to it to form a precrRNA/tracrRNA complex. This complex is further processed to form the mature tracrRNA-crRNA complex known as gRNA. The gRNA will then bind to Cas9 and direct Cas9 to the target site for DNA cleavage to generate DSBs. Every single strand is cleaved by a different structural domain of Cas (17). Whether the intended site can be targeted depends on a short sequence called the Protospacer Adjacent Motif (PAM) located downstream of the intended target site. PAM which controls the targeting specification is recognized by Cas9 and is specific to each subgroup of the CRISPR-Cas9 system (18, 19).

Finally, cutting target DNA to produce DSBs by the nuclease Cas9 initiates the host self-repair machinery, including nonhomologous end joining (NHEJ) and homology-directed repair (HDR) (20). These two restoration mechanisms mediate mutations such as substitutions, deletions, and insertions in the target DNA (17, 21, 22).

Off-target effects are one of the biggest problems facing CRISPR-Cas9 in clinical applications. A study demonstrated that Cas9 nucleic acid endonuclease has high activity even in the presence of gRNA bootstrap mismatch, which would greatly reduce the safety of its clinical application (23). But it has been demonstrated that CRISPR-Cas9 has higher cleavage efficiency and lower off-target rates than TALEN and ZFN gene therapy technologies in the treatment of HPV infection (24). On the other hand, it has been reported that off-target can be minimized by optimizing gRNA sequences as well as Cas9, among other ways (25). For example, Komor et al. significantly improved targeting rates using Cas9-nickase, a D10 mutant of Cas9 (26). And many CRIPSR-Cas9-based editors such as cytosine base-editors (CBEs), adenine base-editors (ABEs) and Prime-editors (PEs) have also been designed to achieve smaller insertion or deletion of mutations (27). More variants of CRISPR and Cas9 are being investigated to optimize this technique. Therefore, although the clinical application of CRISPR-Cas9 still needs to overcome many difficulties, it is still the optimal choice for gene therapy of HPV virus infection.

To apply CRISPR-Cas9 to tumor therapy, in 2012, a single-guide RNA (gRNA) was first designed to contain all the components needed for prerRNA/tracrRNA to guide the Cas9 nuclease (8). Soon after, in 2014, Zhen et al. reported for the first time that targeting the HPV E6/E7 gene using CRISPR-Cas could inhibit tumor cell growth. Researches on CRISPR-Cas for HPV-associated cancers have increased rapidly since then (28).

## Major targets of CRISPR-Cas9 application in HPV cancer

**Figure 2:**
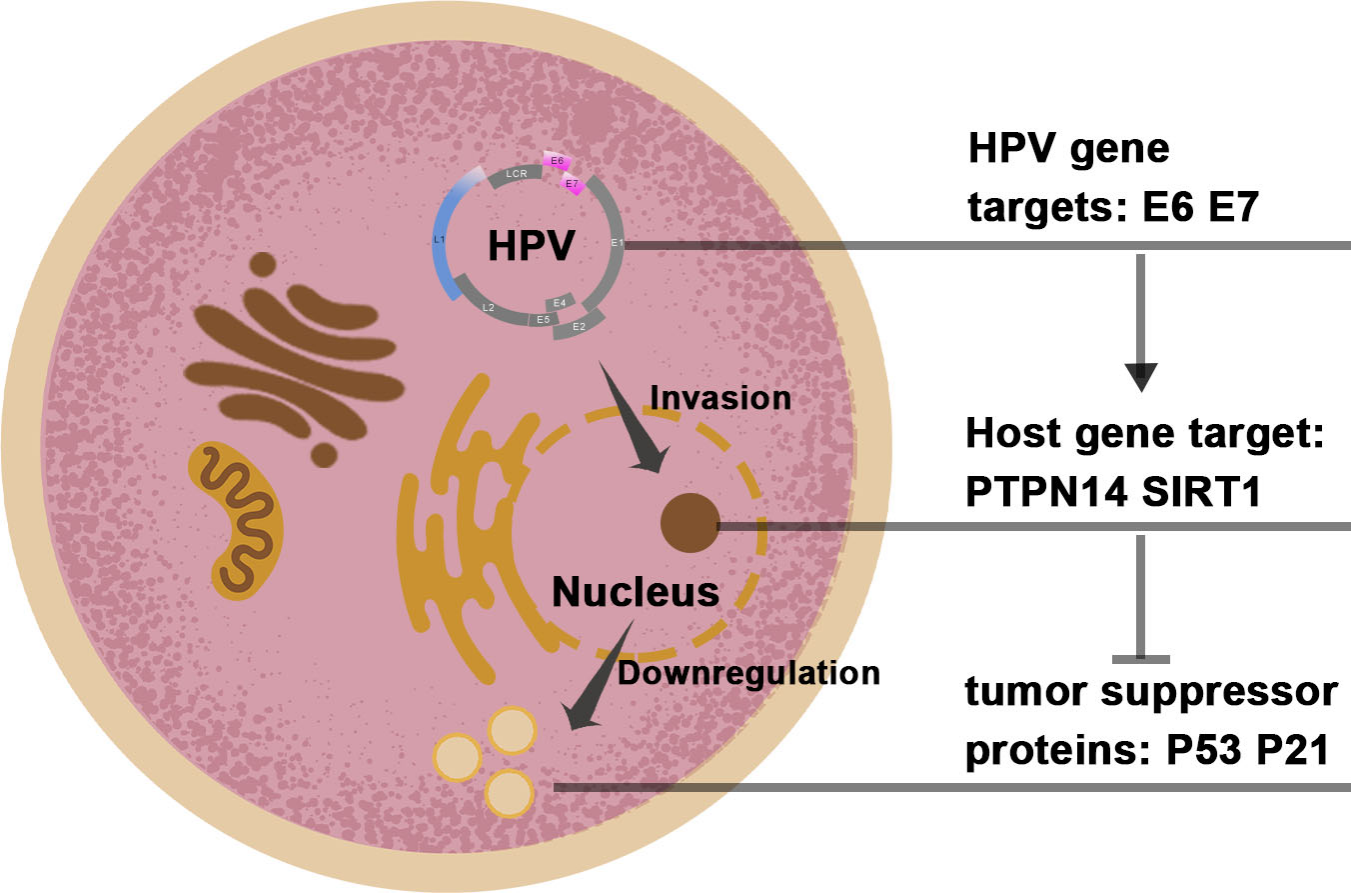
The target genomes in the HPV and host cell.

Studies have proven that over 99% of cervical cancers are directly related to high-risk HPV infection (29). Two subtypes, HPV16 and HPV18, are predominant. The genome of the HPV virus consists of two main parts. The first part is the early region (E), whose main function is to participate in the regulation of virus replication and life cycle; the other part is the late region (L), whose main function is to encode the capsid protein that forms the virus (30). In the genome of HPV, the E6 and E7 genes are the major oncogenes. The E6 oncogene functions by inhibiting the p53 cancer suppressor pathway and blocking the RIG-I signaling pathway (immune escape mechanism), and the E7 oncogene suppresses retinoblastoma protein (Rb) and affects p21 and other pathways (31–33). (**Figure 2**) A growing number of studies have demonstrated that the E6 and E7 genes play a critical role in enabling the induction of apoptosis and cell cycle arrest (34). Therefore, the E6 and E7 genes have also been widely studied by many scholars as to the main target loci (**Table 1**).

**Table 1:** Summary of studies of CRISPR-Cas9 targeting HPV genes.

Editing system & genotype	Target gene	Cell/Animal model	Author/year
CRISPR-Cas9 HPV16	E7	SiHa and Caski cells	(31)
CRISPR-Cas9 HPV16	E6 E7	SiHa and C33A cells	(28)
CRISPR-Cas9 HPV6/11	E7	Human keratinocytes	(35)
CRISPR-Cas9 HPV16	E6 E7	SiHa and C33A cells	(36)
CRISPR-Cas9 HPV16	SIRT1	C33A cells	(37)
CRISPR-Cas9	DNAJA4	HaCaT cells	(38)
CRISPR-Cas9 HPV18	E6 E7	Hela cells	(39)
CRISPR-Cas9 HPV18	E6	HeLa, HCS-2, SKG cells and human immortal cell line 293	(40)
CRISPR-Cas9 HPV16/18	E6 E7	HeLa, CasKi, HEK293T, Jurkat cell lines and HeLa FLAG16E7MYC44	(41)
CRISPR-Cas9 HPV16	SAMHD1	N/Tert-1 cells	(42)
CRISPR-Cas9 HPV16/18	THZ1	HeLa, SiHa, C33A cells and human embryonic kidney cells 293T	(43)
CRISPR-Cas9	CIB1	Human keratinocyte line NKc2115 and the mouse fibroblast line 3T316	(44)
CRISPR-Cas9 HPV16	PIM1	Human HNSCC cell lines FaDu, SCC-4, SCC-9, SCC-15, CAL 27, Detroit 562, SCC-25, UM-SCC-47 and UM-SCC-104	(45)
CRISPR-Cas9 HPV16	E6 E7	SiHa cells	(46)
CRISPR-Cas9 HPV16	WRN	N/Tert-1 and HPV16 cells	(47)
CRISPR-Cas9 HPV18	E6	HeLa and Hek-293 cell lines	(48)
CRISPR-Cas9 HPV16/18	E6 E7	SiHa cells	(49)
CRISPR-Cas9 HPV18	E7	Human foreskin keratinocytes (HFKs)	(50)
CRISPR-Cas9 HPV16	E7	HeLa, UDSCC2 (SCC2),UMSCC104 (SCC104),UMSCC1 (SCC1),FaDu and Detroit 562 cells	(51)
CRISPR-Cas9 HPV18	E6 E7	HeLa cells	(1)
CRISPR-Cas9 HPV16	E7	SiHa, HeLa, CaSki and the HEK cell line HEK293	(52)

In 2014, HPV16-E7 was first recognized as a goal of motion of the CRISPR-Cas9 machine for gene remedying HPV virus-positive cervical cancer (31). In the same year, another study also demonstrated that the CRISPR-Cas9 system targeting the E6 and E7 loci led to a significant accumulation of p53 and p21, which significantly reduced the proliferation of cervical cancer cells *in vitro*, and this finding was also demonstrated *in vivo* in a mouse model (28). In subsequent studies, it was found that knocking out the E6 and E7 genes could inhibit cervical cancer cell proliferation, in addition to several other effects. In 2016, a study confirmed that focused inactivation of the HPV16 E6/E7 gene might also be a high-quality sensitizer of CDDP chemotherapy in cervical cancer (36), providing new ideas for additional gene therapy strategies. The same research team again found that blocking the PD-1 pathway and the HPV16 E6/E7 gene may have a synergistic effect and together enhance the antitumor effect in 2019 (46).

In addition to the treatment targeting the E6/E7 gene, some scholars have also turned their attention to investigating other HPV oncogenic mechanisms using the CRISPR-Cas9 system. In 2017, the SIRT1 gene was knocked out by CRISPR-Cas9, demonstrating the important regulatory role of the cytosolic enzyme SIRT1 in HPV16 replication (37). Subsequently, in 2020, the same research team continued to investigate the regulatory role of the SIRT1-WRN axis using CRISPR-Cas9 technology and noted the dependence of the viral replication cycle on WRN (47). Some scholars have also started to study the host proteins that interact with the transcription products of E6/E7 genes. For example, in 2020, a study found that binding of the E7 oncoprotein of HPV16 and HPV18 to the host tumor suppressor PTPN14 would inhibit the expression of differentiation genes and demonstrated experimentally that mutating the PTPN14 gene in cervical cancer cells by CRISPR-Cas9 would significantly reduce the oncogenic activity of HPV viruses (50). Many studies on CRISPR-Cas9 system-targeted gene therapy are underway.

## Application of CRISPR-Cas9 in HPV cancer therapy

As a therapeutic technique, gene therapy can work by replacing specific molecular defects of genes that contribute to the development or progression of cancer. This therapy has been widely applied, including in cardiovascular diseases, vaccination, and cancers in which conventional therapies have failed. For HPV-driven cancer, various gene therapy approaches have been developed and verified. CRISPR-Cas9, unlike traditional gene-editing technology, can provide an easy way to edit specific sites in the genome and thus offers tremendous opportunities for more diseases. We briefly summarized the CRISPR-Cas9 in various potential applications in HPV (**Table 2**). Detailed information will be introduced below.

**Table 2:** Summary of application of CRISPR-Cas9 in HPV-driven cancer.

Category	Description of CRISPR-Cas9 function	Model of CRISPR-Cas9 medication	Author/year
Interference in HPV	HPV16-E7	HPV16-infected cervical cancer cell lines and HPV16 transgenic mice;	(31, 52-54)
	HPV16-E6	HPV16-infected cervical cancer cell lines	(55)
	HPV16-E6/E7	HPV16-infected cervical cell line and mouse tumor model; HPV16 transgenic mice	(28, 56)
	HPV6/11-E7	E7-transfromed keratinocytes	(35)
	HPV18-E6/E7	E6- or E7- transfected Hela cell; HPV18-infected cervical cancer cell lines (HeLa, HCS−2, SKG−I, CaSki, and SiHa)	(39, 40, 57)
	HPV18-E6	HPV18-E6-infectied HeLa cell	(58)
Manipulate cancer genome	JunB	HNSCC cell lines and lung metastatic mouse model of HNSCC	(59)
	CD55	Cervical cancer cell lines (C33A, C4-1, CaSki, ME180, MS-751 and SiHa)	(60)
	CD71	Cervical cancer cell lines (C33A, C4-1, CaSki, and SiHa)	(61)
	CDK7	Cervical cancer cell lines (HeLa, SiHa, and C33A) and subcutaneous xenograft mouse model	(43)
	PIM1	Human HNSCC cell lines and mouse xenograft model	(62)
	p53	HPV16-transformed human keratinocytes (HKc/DR)	(63)
Enhance immunotherapy	The immune checkpoint PD1	HPV16-infected cervical cancer cell lines and HPV16 transgenic mice	(46)
	Combinatorial therapy of GSK126, an EZH2 inhibitor, and anti–PD-1	Human HNSCC cell lines and mice model	(64)
Effects on the other therapies	An effective sensitizer of CDDP chemotherapy	SiHa cells and xenograft mouse models of cervical cancer	(36)
	Optimal radiosensitization approaches	Virous cell lines and xenograft models	(65)
	MLL5 genes on chemotherapy	HeLa and Hek-293 cells	(48)
Delivery system of CRISPR-Cas9	Plasmid	Cells (CasKi, HPV16 positive; HeLa, HPV18 positive); xenograft mouse model	(41, 66)
	AAV vector.	HPV18-infected cervical cancer cell lines (HeLa, HCS−2, SKG−I); HPV18-positive HeLa cell line	(40, 58)
	Nanoparticle (NPs)	Cervical cancer cells lines; xenograft tumors in mice model	(52, 67, 49)
	High-capacity adenoviral (HCAdV)	Cervical carcinoma cell lines (HeLa, CaSki, and SiHa)	(57, 68)
	HPV pseudotype virus	Cervical cancer SiHa cells and nude mice model	(69)
	Endogenous exosomes-mediated delivery	Hela cell	(70)
	Liposome	HPV16-infected cervical cell line and HPV16 transgenic mice; cervical cancer cell lines (Hela and SiHa) mouse tumor model	(46, 49, 54)
Detection	CRISPR- or Cas9/gRNAs-associated reverse PCR(CARP)	HPV-positive cervical carcinoma cells (HeLa and SiHa)	(71)
	CRISPR-typing PCR(ctPCR)	Human cervical carcinoma cells (SiHa, HeLa and C-33a)	(72, 73)
	CRISPR-Cas9-assisted DNA detection (CADD)	Human cervical carcinoma cells (SiHa, HeLa and C-33a)	(74)

### CRISPR-Cas9 technology applications for HPV

**Figure 3:**
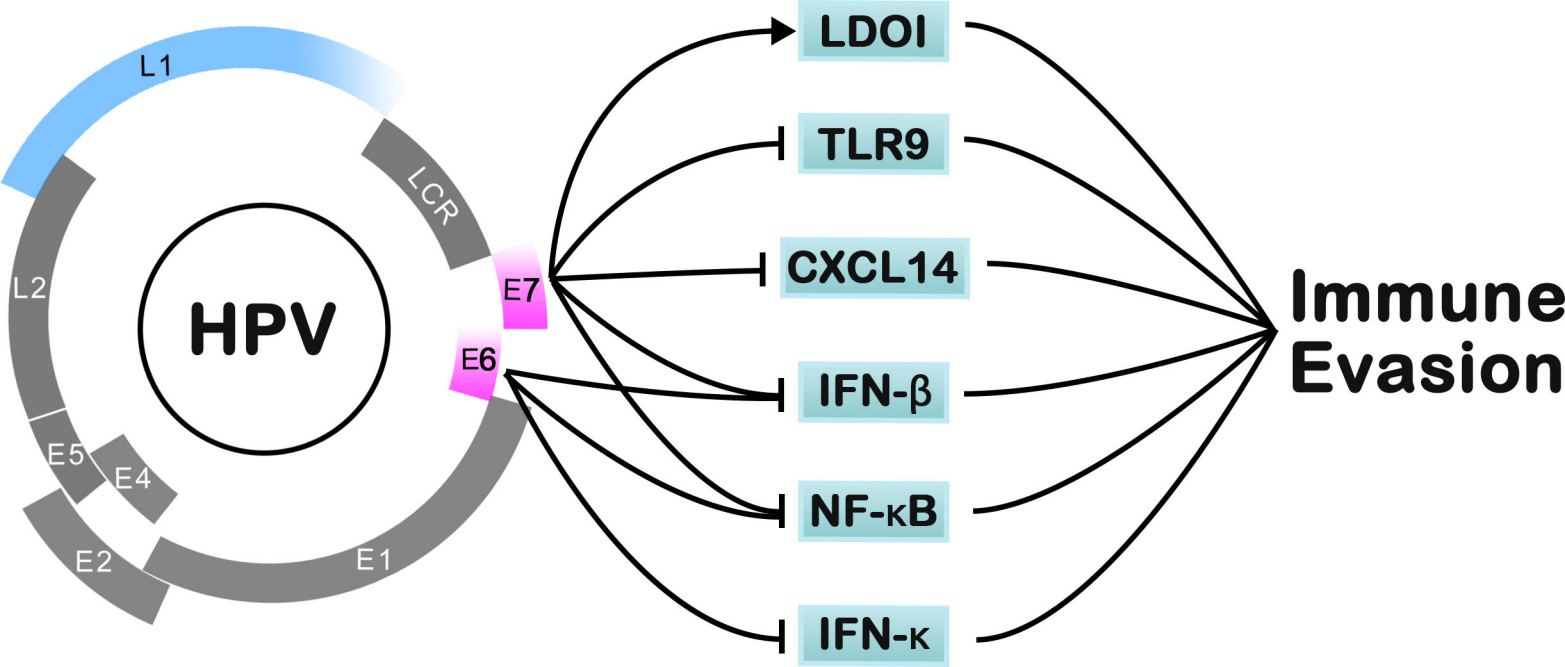
The pathways of HPV for immune-evasion mechanisms.

There is a common etiologic feature in HPV-driven cancer that the emergence and function of viral oncogene expression (E6/E7) are related to the tumor cells, far away from stromal cells. Relevant research confirmed that most HPV-driven cancers can classify the oncogenic proteins E6/E7 to inactivate the host tumor suppressors p53 and RB, respectively. In the process of cell oncogenesis, E6 and E7 promote the replication of the viral genome and induce malignant biological properties, including uncontrolled cellular proliferation, angiogenesis, invasion, and metastasis (75) (**Figure 3**). A previous study showed that E6/E7-inactivated HeLa cells displayed distinctive senescence markers, such as an enlarged cell surface area (1).

E6/E7 and their complex have deserved as specific targets in gene therapy. Several strategies targeting E6/E7 have been reported, including cytotoxic drugs and zinc-ejecting inhibitors of the viral E6 oncoprotein (76–78), E7 antagonist peptide (79), and HSP90 and GRP78 inhibitors targeting E6 and E7 (80). Recently, CRISPR-Cas9 has entered into clinical trials as a novel therapeutic strategy. The therapeutic mechanism of CRISPR-Cas9-mediated downregulation of E6 and E7 normally attributes to the inactivation of p53 and RB to set off apoptosis and mobile senescence.

High-risk HPVs (such as HPV-16 and HPV-18) can encode viral oncoproteins E6 and E7 in tumor cells, most common in cervical and penile cancers. The targeted gene by CRISPR-Cas9 performed well in a large number of studies. In 2014, the first CRISPR-Cas9 system featuring HPV16-E7 single-guide RNA (gRNA) showed disruption of HPV16-E7 DNA at specific sites induced apoptosis and growth inhibition of HPV-driven cancer cells (31). Further study conducted gene therapy in the K14-HPV16 transgenic mouse model, which had a favorable effect on cervical precancer (53). In 2015, the HPV16-E6 oncogene was cleaved by the customized CRISPR gRNA/Cas9, which demonstrated for the first time that it could be a therapeutic approach to reverse the malignant phenotype and increase the expression of p53 (55). Both viral oncoproteins are therefore regarded as promising targets for gene therapy. The knockdown of E6/E7 has been pronounced in *in vivo* and vitro trials alongside an accumulation of p53 and p21 protein and should result in remarkably issue of the proliferation of HPV-driven most cancers cells (36, 56).

In addition, other HPV types were also studied for their biological properties and use in genetic therapy with CRISPR-Cas9. As an HR-HPV, HPV-18 has been targeted and tested *in vitro* and *in vivo*. Previous studies showed that oncogenes E6 and E7 of HPV18 could be successfully inhibited by the CRISPR-Cas9 system (39, 40, 57, 58). However, HPV-6/11, which are low-risk HPV types most common in anogenital warts and laryngeal papillomatosis, also encode the oncoproteins E6/E7. Thus, a CRISPR−Cas9 system centered on HPV6/11 has been stated as a novel and fairly effective molecular purpose for the treatment and prevention of low-risk HPV-driven diseases (35).

With the deepening look up on HPV, more gene sequences will flip out to be ambitions of gene treatment for HPV illness or remedy of HPV-driven cancer. CRISPR-Cas9 can additionally be used in the preparation of vaccines to prevent HPV infection (81). Nonetheless, the pleasant transport of developed Cas9 plasmids *in vivo* remains a challenge. Further look up should be carried out to transport Cas9 and gRNA into the intention cell of the human physique and to make positive the biosafety need.

### Manipulation of the cancer genome by CRISPR-Cas9

As a promising progressive technological know-how in gene editing, CRISPR-Cas9 provides scientists with a number of alternatives to manipulate the genome of cancers and trade the DNA structure.

Oncogenes are a group of mutated genes that may cause cancer, such as JunB and PIM1. JunB is a unique factor of activator protein-1 transcription factors, appearing both as a tumor suppressor or as an oncogene relying on the cell context (82). JunB knockdown and knockout limited the progression of tumor migration and invasion, suggesting that the downregulation of JunB expression might be a potential therapeutic strategy for inhibiting distant metastasis in patients with HPV-driven cancer (59). Another oncogene, PIM1, encoding a constitutively active serine/threonine protein kinase, was investigated for its functional roles in the viability and growth of HPV-driven cancer cells (83). CRISPR-Cas9-mediated exchange of PIM1 resulted in cell cycle arrest and apoptosis in HPV-driven most cancers (62). In addition, the genetic depletion of CDK7 using the CRISPR−Cas9 system exhibited great cell growth inhibition in cervical cancer cell lines (43). Additionally, HPV16-transformed cells with CRISPR−Cas9-mediated loss of p53 were inclined to lose dependence on the continuous expression of HPV oncogenes for proliferation (63).

The identification of cell surface markers in cancer can establish differentiation to target specific sites. Some research confirmed a novel affiliation between HPV-E6 oncoprotein expression and the increase in the CD55 and CD71 floor markers in most cervical cancer cells (60, 61). The HPV-E6 oncoprotein enriched the CD55 and CD71 populations, which increased cell proliferation, cell self-renewal ability, cell migration, radioresistance, and tumorigenicity. The knockdown or knockout of CD55 and CD71 expression in HPV-E6-expressing cells should reverse the tumorigenic phenotypes of most cervical cancer cells.

HPV plays a role in the pathologic process of HPV-driven cancers. This finding suggests that HPV prophylactic vaccines will have a wider range of protection, making us far from a series of HPV-driven cancers, especially in head and neck cancer and cervical cancer, where the expression of HPV is relatively high.

### Enhanced immunotherapy with CRISPR-Cas9

With the advent of immune checkpoint inhibitors (ICIs), immunotherapy has emerged as one of the most promising therapeutic strategies for cancer. CRISPR−Cas9, as a versatile and easily used genetic enhancing technology, is frightening a progressive change in most cancer immunotherapies. Recently, CRISPR−Cas9 has been carried out to incite the improvement of therapeutic immune agents, such as chimeric antigen receptor T (CAR-T) cells and the programmed cell dying protein-1 (PD-1) or its ligand (PD-L1) (84).

PD-1 and PD-L1 are negative regulators of the immune responses of T cells (85). Expressed on cervical T cells and DCs. PD-1 and PD-L1 have been lately pronounced to be related to excessive risk-HPV positivity and to be increased along with growing cervical intraepithelial neoplasia (CIN) grade (86). Furthermore, the researcher established a C33 cell line stably expressing HPV16 E6/E7 (SiHa-E6/E7) to verify the promotive association between HPV16 E6 and the expression of PD-1. Based on this, they tested whether combined targeted therapy with immunotherapy can 1 + 1 equal more than 2 in the SiHa cervical cancer mouse model. Synergistic effects have been reported for combination therapy targeting HPV16 E6/E7 and PD-1 blockade using CRISPR-Cas9 (46).

EZH2, a catalytic subunit of polycomb repressive complex 2 (PRC2), was reported to block PD-1/PD-L1 axis downregulation. High expression of EZH2 was also associated with tumor cell proliferation, invasion, and metastasis and has important clinicopathologic significance (87). EZH2 knockdown or inhibition has been tested in mice with induced endometriosis and prompted EZH2-induced epithelial-mesenchymal transition (EMT) in cancers (88). Further research identified EZH2 as a potential therapeutic target for encouraging antigen presentation and antitumor immunity in head and neck squamous cell carcinoma (HNSCC) (64). The combination of EZH2 inhibition and anti-PD-1 therapy may be beneficial for patients with HNSCC, which requires further preclinical studies.

Academic clinical trials have investigated T cells with PD-1 knocked out by CRISPR-Cas9 for the treatment of multiple types of cancer. Among the trials, it has recently attained a significant therapeutic effect on NSCLC (10). However, we have not found any research directly exploring novel and promising cancer immunotherapy with the knockout of CAR-T or PD-1 by CRISPR−Cas9 in HPV-driven cancers.

### Effects of CRISPR-Cas9 on other therapies

For patients with HPV-driven cancers, conventional therapy includes chemotherapy, radiotherapy, or subsequent chemoradiotherapy (CCRT). One of the major issues in clinical oncology is the ability of cancer cells to resist chemotherapy drugs, which leads to chemotherapy failure.

In 2016, the first record of HPV16 E6/E7 focused on CRISPR-Cas9 was published, in which the method was once described as a nice sensitizer for bettering CDDP chemotherapy in cervical cancer (36). It ought to efficiently and especially coordinate with CDDP for HPV16 fantastic cervical cancer. This further indicated the position of the blended lineage leukemia 5 (MLL5) in the carcinogenesis of most HPV-positive cervical cancer cells (48). Knockout of MLL5 greatly impacted the chemotherapeutic effectivity of cisplatin in HPV-18-positive cells. Additionally, the finding that MLL5 has a higher anticancer effect than E6 by means of CRISPR-Cas9 has an impact on the disruption of MLL5.

For head and neck squamous cell carcinoma (HNSCC), radiotherapy is one of the most commonly used and effective treatments. However, different HPV genotypes of HPV-driven cancers, such as cervical cancer patients with HPV-18 DNA, have significantly different responses to radiotherapy (89). Recently, a novel screen based on a targeted CRISPR−Cas9 system was applied to identify optimal radiosensitization approaches for HPV-positive/negative HNSCC (90). The combination of radiotherapy and CRISPR−Cas9-mediated inhibition of genetic repair pathways could improve the therapeutic response in patients with HNSCC.

### HPV detection with CRISPR-Cas9

Nucleic acid detection techniques are always crucial to diagnosis, especially in the background of the present coronavirus disease 2019 pandemic. Despite a wide range of applications of genetic testing tools, the CRISPR-Cas9 system has advantages in the detection field and has been applied in gene editing and regulation. We know that polymerase chain reaction (PCR) is widely applied due to the high sensitivity of the exponential amplification of target DNA. Therefore, PCR has been the most popular DNA detection and genotyping technique, such as detecting SARS-CoV-2 and diagnosing the current COVID-19 pandemic. To date, the combination of PCR and CRISPR techniques provides a new chance for developing new nucleic acid detection and typing techniques.

In 2018, Qiao and Beibei first developed CRISPR-Cas9-associated reverse PCR (CARP), in which Cas9-cut target DNA was cyclized and detected by reverse PCR amplification (71). Due to the reverse PCR amplification of the DNA of interest that was performed in detection, this method had high sensitivity. Based on the technique, they then developed a new method for detecting and typing target DNA based on Cas9 nuclease, which was named ctPCR (72). By using qPCR machines, the whole ctPCR detection process can be finished in as little as 3 to 4 hours. Thus, ctPCR should be useful in DNA detection and genotyping. However, the target DNA was detected and genotyped based on comparing the Ct values and DNA copies of two qPCRs. Afterward, a new version of ctPCR was developed to avoid this comparison step, which symbolized one-pot detection (73). The whole detection process can be finished on PCR instruments without further tube opening.

Outside of the PCR technique, another CRISPR-Cas9-mediated DNA detection method called CRISPR−Cas9-assisted DNA detection (CADD) was developed (74). The detection of target DNA could be completed in less than 30 min, according to the unique advantages over current methods, such as being simple, rapid, and free of preamplification and the application of fluorescent hybridization chain reaction (HCR).

### Delivery system of CRISPR-Cas9 for HPV

The delivery system of CRISPR-Cas9 applied in the human body remains a challenge. Even when many specific molecular targets are available to select for tumor cells, it is quite controversial to identify an effective and safe transport system. To overcome this issue, researchers have exploited several kinds of carriers, from viral delivery systems to liposomes and from plasmids to nanoparticles. We discussed the pros and cons of each delivery system based on current research reports.

Among the viral shipping vectors, high-capacity adenoviral vectors (HCAdV) have the potential for packaging up to 35 kb, permitting handing over the entire CRISPR-Cas9 equipment inclusive of numerous gRNAs (68). In contrast to adenoviral vectors (AdV), they had no threat of expressing AdV genes with much less immunogenic properties (91). The proof-of-concept for the use of CRISPR-Cas9 delivered by the most superior adenoviral vector (HCAdV) has a considerable impact in treating HPV-derived tumors (57). Nevertheless, manufacturing of HCAdV is intensive in time and work when compared with AAV-vector platforms, hampering their exploration for unique applications. Different from the normal viral shipping system, AAV-based shipping structures have shown predominant benefits (92), which have attracted much attention, especially for therapeutic purposes. Additionally, AAV can have stable transgene expression with long-term existence as a concatemer in nondividing cells (93). The AAV-based CRISPR-Cas9 machine has been used for disruption of the E6 gene in HeLa, which emphasized AAV-based viral vectors as one of the most sensitive viral vectors for gene remedy and gene switch *in vivo* (40, 58).

However, the viable cytotoxicity, immunogenic response, and long-term expression of viral vectors continue to be issues of scientific application (94). Several nonviral transport techniques have been reported. In 2019, a study indicated the viability of endogenous exosomes as a protected and fantastic transport carrier of the purposeful gRNA and Cas9 protein (70).Scientific hobby in nanoparticles (NPs) is on the rise due to their versatility and, in particular, their large applicability (95). A study has developed NPs consisting of PBAE546 and CRISPR-Cas9 for the treatment of HPV infection, which provides new hope for the clinical transformation of nanomedicine to treat cervical lesions, thereby preventing cervical cancer (52). Other research also confirmed this genetic strategy effectively (49, 67, 96).

## Conclusion

CRISPR-Cas9 technology presents a new device for the genetic detection and remedy of most HPV-driven cancers from a specific aspect. With numerous benefits over traditional methods, such as being simple to design, easy to use, and efficient to edit, the remedy affords a promising method for medical applications. However, nearly complete gene treatment plans associated with CRISPR-Cas9 continue to be in the experimental phase, with current off-target consequences and other safety perils. Collectively, the novel genetic cure of HPV-driven most cancers can be anticipated with the leap forward of CRISPR-Cas9 technology.

## Author contributions

YW and ZZ contributed equally to this work. YW and ZZ participated in the literature search and data collection. YW wrote the manuscript in consultation with ZZ. XM participated in the study conception and manuscript revision. All authors discussed the results and contributed to the final manuscript.

## Conflict of interest

The authors declare that the research was conducted in the absence of any commercial or financial relationships that could be construed as a potential conflict of interest.

## Publisher’s note

All claims expressed in this article are solely those of the authors and do not necessarily represent those of their affiliated organizations, or those of the publisher, the editors and the reviewers. Any product that may be evaluated in this article, or claim that may be made by its manufacturer, is not guaranteed or endorsed by the publisher.
